# Emerging Roles of Regulated Cell Death-mediated Inflammation in Pathophysiology of Ocular Diseases

**DOI:** 10.18502/jovr.v21.20210

**Published:** 2026-05-24

**Authors:** Nader Sheibani

**Affiliations:** ^1^Department of Ophthalmology and Visual Sciences, University of Wisconsin School of Medicine and Public Health, Madison, USA; ^2^Department of Cell and Regenerative Biology, University of Wisconsin School of Medicine and Public Health, Madison, USA; ^3^McPherson Eye Research Institute, University of Wisconsin School of Medicine and Public Health, Madison, USA

**Keywords:** A Apoptosis, Cuproptosis, Diabetic Retinopathy, Ferroptosis, Glaucoma, Necroptosis, Ocular Surface Diseases, PANoptosis, Pyroptosis

## Abstract

Regulated cell death pathways are vital for proper developmental and homeostatic processes. Dysregulation of these pathways contributes to the pathogenesis of many diseases, including ocular inflammatory and neurodegenerative diseases such as glaucoma, diabetic retinopathy, age-related macular degeneration, retinitis pigmentosa, and ocular surface diseases. Our knowledge of the regulated cell death pathways, including apoptosis, necroptosis, pyroptosis, and ferroptosis, has been extensively expanded in the recent years. The targeting of these pathways as a potential therapy for various ocular diseases is now widely recognized. In recent years, it has also become clear that in many circumstances, the engagement of multiple regulated cell death pathways could be coordinated through specific cross talks to drive a stress-specific cell death and disease pathogenesis. This knowledge is extended to the recognition that targeting multiple regulated cell death pathways could be more effective for the treatment of various pathologies. However, the identity of upstream regulatory pathways and the engagement hierarchy of individual pathways and their coordinated interactions require further investigation. Here, I will briefly introduce these regulated cell death processes, discuss the key regulatory pathways involved in determining cell death or survival, as well as upstream modulators. I will also highlight studies targeting these pathways as potential treatment strategies for various eye diseases.

##  INTRODUCTION

**Figure 1:**
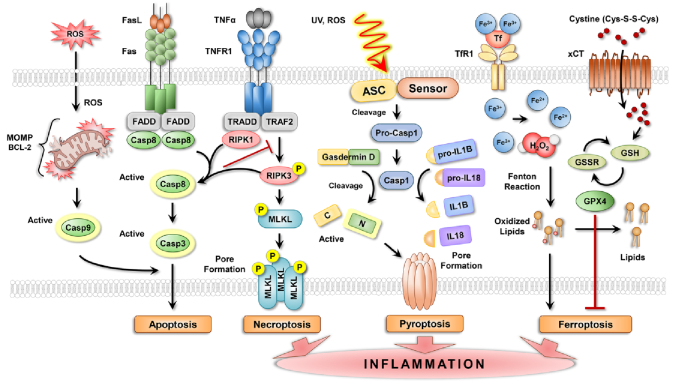
Schematic representation of regulated cell death pathways. Apoptosis can proceed through either the intrinsic (mitochondria-mediated) or extrinsic (death receptor-mediated) pathway involving FAS or TNFR1 activation, typically without triggering inflammation. In contrast, regulated necrotic pathways—including necroptosis, pyroptosis, and ferroptosis—lead to inflammatory cell death. Necroptosis is initiated by TNFR1 activation, resulting in phosphorylation of RIPK3 and MLKL. Oligomerized pMLKL forms pores in the plasma membrane, leading to membrane rupture and necroptotic death. Pyroptosis involves inflammasome formation and activation of procaspase-1, which cleaves Gasdermin D and pro–IL-1 β /IL-18. The N-terminal fragment of Gasdermin D forms membrane pores, promoting the release of IL-1 β and other proinflammatory mediators that amplify inflammation and cell death. Ferroptosis is driven by iron-dependent Fenton reactions and lipid peroxidation. The antioxidant enzyme GPX4 inhibits lipid peroxidation and protects against ferroptosis. The image was adapted from Upadhyay and Bonilha.^[96]^ MOMP, mitochondrial outer membrane permeabilization;FADD, Fas-associated death domain; TRADD, TNF receptor-associated death domain; TRAF2, TNF receptor-associated factor 2; GR, glutathione reductase; ASC, apoptosis-associated speck-like protein containing a caspase recruitment domain. Other abbreviations are defined in the text.

Apoptosis, or programmed cell death, was the first known cell death pathway that was recognized to be actively regulated. Apoptosis generally occurs through two major pathways, the mitochondrial-dependent or “intrinsic” and mitochondrial-independent or “extrinsic” pathways, regulated by a specific family of proteins. The intrinsic pathway is regulated by the Bcl-2 family of proteins with pro- and anti-apoptotic activities, whose main function is to maintain the integrity of mitochondrial function through domain-specific protein–protein interactions among different family members.^[1,2,3,4,5]^ The extrinsic pathway involves signaling through death receptors and a family of adaptor proteins whose engagement through death factors such as tumor necrosis factor α (TNF α ; also a proinflammatory mediator) leads to the formation of a death-initiating complex.^[6,7]^ Both pathways ultimately utilize a family of aspartate-specific cysteine proteases downstream known as caspases or executioners [Figure 1].

Caspases are important in amplifying the death signals and mediate the degradation of intracellular constituents, and finally drive the cell's demise^[8]^. However, the intracellular components are maintained within the boundary of the cytoplasmic membrane, and as a result, no inflammation is associated with these apoptotic cell death pathways. In contrast, necrosis is the other major form of cell death that was initially thought to be passive and not regulated, resulting from overwhelming cellular stress or mechanical forces. As a result, the cell's plasma membrane ruptures, and the release of cellular contents results in activation of the innate immune response, aiding the induction of the proinflammatory cytokines and chemokines, likely through coordinated actions of an extensive array of specific sensors, adaptors, and effector proteins.

In the past two decades, a group of proteins has been identified that can regulate necrosis pathways. The widely studied regulated forms of necrosis include necroptosis, ferroptosis, and pyroptosis. The activation of these pathways, detailed below, through a specific set of proteins initiates programmed necrosis pathways terminating with a compromised plasma membrane and release of cellular content, ultimately resulting in engagement of inflammatory processes and inflammation.^[9,10,11,12,13]^ It is also now being recognized that the sequential activation of multiple regulated cell death pathways, including pyroptosis, apoptosis, and necrosis, can be activated, whose integrated activities modulate cell death, which is referred to as PANoptosis.^[14,15,16]^ The details of these mechanisms and their hierarchy in promoting cell death remain largely unknown and could be influenced by the types of insults cells encounter. In the next section, I will introduce the different forms of regulated cell death pathways, focusing on necrotic pathways since apoptotic pathways have been extensively studied and previously discussed in numerous publications and reviews.^[6,17,18,19]^ However, various components of apoptotic pathways, including caspases, could make a significant contribution to the coordinated activities of these programmed necrosis pathways.

##  REGULATED NECROSIS CELL DEATH PATHWAYS 

Although necrosis was initially considered a passive form of cell death, studies in the past two decades have identified highly regulated and genetically controlled pathways that modulate different forms of necrosis in response to various stress stimuli. The regulated necrosis cell death pathways include necroptosis, pyroptosis, ferroptosis, oxytosis, parthanatos, PANoptosis, and cuproptosis. These pathways are engaged under specific stress conditions through interactions among a group of functionally related proteins that ultimately lead to the death of damaged cells in a caspase-dependent and/or independent manner. Furthermore, multiple forms of these regulated necrosis pathways may occur concurrently, whose specific activation is coordinated through specific sensors and interactions among a set of regulatory proteins from these pathways. Understanding how these pathways are initiated and could be integrated to drive the cell demise in response to a specific stress signal remains an area of significant research interest.

### Necroptosis

Necroptosis is a regulated form of cell death and morphologically is similar to necrosis (cell swelling, plasma membrane rupture, and inflammation).^[20,21]^ However, unlike accidental necrosis, it is tightly controlled by a specific set of proteins that initiate and propagate death signals. It serves as an alternative cell death mechanism when apoptosis (the more controlled, noninflammatory form) is inhibited, often during viral infection, inflammatory diseases, or certain types of tissue injury. Necroptosis can function as a backup defense mechanism against pathogens that block apoptosis, ensuring infected or damaged cells still die. Cells swell, organelles dilate, plasma membrane integrity is lost, and intracellular contents are released, which trigger the host's innate immune response and result in inflammation. It is different from apoptosis. Apoptosis is caspase-dependent and noninflammatory, while necroptosis is caspase-independent (especially caspase-8) and pro-inflammatory.^[22,23]^

The necroptosis pathway is most often initiated by death receptors such as TNF receptor 1 (TNFR1), but also by pattern recognition receptors (PRRs) such as TLR3, TLR4, and interferon receptors.^[22,24]^ The key mediators of necroptosis include Receptor-Interacting Serine/Threonine-Protein Kinase 1 (RIPK1), which functions as a platform protein downstream of TNFR1. Although its global deletion in mice is developmentally lethal, its kinase-dead mutants are viable but exhibit various defects.^[25]^ For example, its kinase activity is critical for necroptosis initiation.

Caspase 8 is another key player in switching between necrosis and apoptosis.^[26]^ When caspase-8 is inhibited, RIPK1 shifts from promoting apoptosis to assembling the necrosome. Receptor-Interacting Serine/Threonine-Protein Kinase 3 (RIPK3), which is activated by RIPK1 or independently via some PRRs, phosphorylates Mixed-Lineage Kinase Domain-Like Protein (MLKL), the executioner of necroptosis and the central hub in necroptotic signaling.^[27]^ Upon its phosphorylation by RIPK3, MLKL oligomerizes and translocates to the plasma membrane and forms disruptive pores in the membrane, leading to cell rupture.^[28,29]^ The interaction between RIPK3 and Caspase 8, and changes in their expression levels and activities, are important in driving downstream events leading to necrosis or apoptosis [Figure 1].^[26,27,30]^ We recently showed the significance of these interactions in mouse retinal vascular development and neovascularization.^[30]^

In summary, necroptosis can be triggered by TNF- α binding TNFR1 and leads to the formation of complex I, where RIPK1 associates with TRADD, TRAF2/5, and cIAPs (pro-survival signaling). If caspase 8 is inhibited or absent, RIPK1 and RIPK3 form the necrosome, switching to death signaling. This leads to RIPK3 activation, which phosphorylates MLKL, driving its oligomerization and membrane insertion, disrupting membrane integrity, leading to cell lysis and release of Damage-Associated Molecular Patterns (DAMPs), which engage innate immune cells, causing inflammatory responses.

The inhibitors of necrosis include Necrostatin-1 (Nec1; RIPK1 inhibitor); GSK'872 (RIPK3 inhibitor); and Necrosulfonamide (NSA; MLKL inhibitor).^[31,32,33]^ Caspase-8 suppresses necroptosis under normal conditions by cleaving RIPK1/RIPK3. The ubiquitination/de-ubiquitination enzymes also control RIPK1's decision between pro-survival and necroptotic pathways.^[24]^ A recent study also showed that metabolic changes, such as decreased methionine levels, could impact Ripk1 activation through post-translation modification,^[34]^ and the ability to modulate adenosine levels.^[35]^

### Pyroptosis

Pyroptosis is a highly inflammatory form of regulated cell death.^[24]^ It is triggered mainly in response to microbial infection or danger signals. Its hallmark is Gasdermin-mediated plasma membrane pore formation, leading to rapid cell swelling, membrane rupture, and the release of pro-inflammatory cytokines such as IL-1 β and IL-18, thereby setting off an inflammatory alarm [Figure 1].^[24,36]^ The purpose of pyroptosis is host defense; it kills infected cells and alerts the immune system. Morphologically, the cell swells and has large plasma membrane pores, and DNA fragmentation, without nuclear condensation typical of apoptosis. The inflammatory nature of pyroptosis has been linked to massive release of DAMPs and cytokines, often initiated by intracellular PRRs detecting pathogens or cell stress.^[24]^ The key molecular players involved in pyroptosis include the PRRs, which sense pathogen-associated molecular patterns (PAMPs) or DAMPs and assemble inflammasomes. Inflammasomes include the NOD-like receptor family pyrin domain-containing 3 (NLRP3), which is activated by a wide range of signals, including ATP, toxins, crystals, and reactive oxygen species (ROS). The NLR family caspase activation and recruitment domain-containing 4 (NLRC4) inflammasome detects bacterial flagellin or type III secretion system proteins in response to stress during sterile chronic inflammation. The absence of melanoma 2 (AIM2) inflammasome senses cytosolic double-stranded DNA, initiating inflammation to prevent infection. It could also contribute to autoimmune diseases and cancer. The Pyrin inflammasome is activated by certain bacterial toxins and cellular stress. These inflammasomes detect various triggers and are coupled to caspase-1 through apoptosis-associated speck-like protein containing a CARD (ASC). Pro-Caspase-1 is an inactive precursor of the executioner enzyme Caspase-1. In the canonical pyroptosis pathway, Caspase-1, autoactivated in the inflammasome complex, cleaves Gasdermin D (GSDMD), which forms pores in the plasma membrane, and releases the mature form of Pro-IL-1 β , Pro-IL-18, and other inflammatory cytokines.

In the non-canonical pyroptosis pathway, Caspase-4/5 (human) or Caspase-11 (mouse) can directly detect cytosolic lipopolysaccharides (LPS) and cleave GSDMD without a traditional inflammasome. Gasdermin D is the executioner protein in pyroptosis. Its cleavage by inflammatory caspases releases its N-terminal domain, which oligomerizes and inserts into the plasma membrane, forming large pores. These pores allow the cytokine to release and drive osmotic swelling and cell rupture, leading to a strong inflammatory response. The key differences between necroptosis and pyroptosis are summarized in Table 1.

**Table 1:** Key differences in pyroptosis vs. necroptosis

**Feature**	**Pyroptosis**	**Necroptosis**
Executioner	Gasdermin D pores	MLKL membrane rupture
Initiation	Inflammasome sensors	TNF receptor / PRRs
Caspase dependency	Yes (inflammatory caspases)	No (caspase-independent)
Inflammatory	Highly	Highly

### Ferroptosis

Ferroptosis is a distinct, iron-dependent form of regulated cell death driven by the accumulation of lethal lipid peroxides in cell membranes.^[9,10,11]^ Unlike apoptosis, necroptosis, or pyroptosis, ferroptosis is not mediated by caspases, necrosome formation, or Gasdermin. Ferroptosis hallmark is oxidative damage to polyunsaturated fatty acids (PUFAs) in phospholipids [Figure 1].^[37,38,39,40]^ The key morphological features of ferroptosis include shrunken mitochondria with condensed membrane densities; reduced/absent mitochondrial cristae, rupture of mitochondrial outer membrane, and no chromatin condensation as occurs in apoptosis. The biochemical hallmark of ferroptosis includes excess lipid peroxidation that overwhelms the cell's antioxidant defenses. However, it is dependent on redox-active iron for ROS generation via the Fenton reaction and iron-containing enzymes.^[37]^

Ferroptosis occurs when the cellular oxidative balance tips toward lipid peroxide accumulation and antioxidant systems fail to neutralize them. The key molecular players include Iron metabolism that involves transferrin receptor (TFRC, imports Fe³⁺ into the cells), and Ferritin (FTH1/FTL, stores iron), whose degradation via ferritinophagy releases free Fe²⁺, fueling the Fenton chemistry.^[10]^ In the Fenton reaction (Fe²⁺ + H₂O₂ → Fe³⁺ + OH), the production of hydroxyl radicals drives lipid peroxidation. The lipid peroxidation machinery includes Acyl-CoA Synthetase Long-Chain Family Member 4 (ACSL4), which activates PUFA-containing fatty acids for membrane incorporation,^[39]^ Lysophosphatidylcholine Acyltransferase 3 (LPCAT3), which incorporates PUFAs into phospholipids, and Arachidonate Lipoxygenases (ALOXs such as ALOX15), which enzymatically oxidize PUFA-phospholipids into lipid hydroperoxides.

The antioxidant defense systems include the Glutathione Peroxidase 4 (GPX4),^[41]^ the central enzyme that reduces lipid hydroperoxides (PUFA–OOH) to nontoxic alcohols (PUFA–OH), using glutathione (GSH) as a cofactor. GSH is a tripeptide antioxidant synthesized from cysteine, glutamate, and glycine. System Xc⁻ (encoded by SLC7A11/SLC3A2) is the cystine/glutamate antiporter that imports cystine for GSH synthesis.^[42]^ Ferroptosis Suppressor Protein 1 (FSP1/AIFM2) is an alternative GPX4-independent defense that regenerates coenzyme Q10 (ubiquinol) to trap lipid radicals. Dihydroorotate Dehydrogenase (DHODH) has a mitochondrial antioxidant role similar to that of FSP1 in the inner membrane.^[10,43,44]^

The sequence of events that lead to ferroptosis includes the trigger, such as inhibition of GPX4 (using RSL3) or GSH depletion (using Erastin to block system Xc⁻). The inhibition of system Xc ${}^{-}$ results in decreased cystine import, which causes a drop in GSH synthesis. Inhibition of GPX4 activity leads to increased lipid peroxide accumulation. The iron-driven peroxidation using free Fe²⁺ catalyzes ROS generation, accelerating lipid oxidation. When the lethal lipid peroxide threshold is reached, the membrane integrity becomes compromised, leading to cell death. The common ferroptosis inducers include Erastin (inhibits system Xc⁻), RSL3 (direct GPX4 inhibitor), FIN56 (promotes GPX4 degradation), and Buthionine Sulfoximine (BSO) (blocks GSH synthesis). The inhibitors of ferroptosis include Ferrostatin-1 (Fer1), Liproxstatin-1 (a lipid radical scavenger), Deferoxamine (DFO, an iron chelator), and Vitamin E ( α -tocopherol; lipid-soluble antioxidant).

### Oxytosis

Oxytosis is a regulated, oxidative stress-driven form of cell death that is mechanistically similar to ferroptosis but was originally described in neurons.^[45]^ It is triggered by the inhibition of the cystine/glutamate antiporter system Xc⁻, leading to depletion of intracellular cystine and GSH. This depletion disables the cell's antioxidant defense, causing unchecked lipid peroxidation and cell death. The key players in oxytosis include System Xc⁻, which imports cystine in exchange for glutamate. Its inhibition by Erastin or extracellular glutamate accumulation depletes cystine, thereby reducing GSH synthesis. GSH is a major intracellular antioxidant, and low GSH levels impair ROS detoxification. GPX4 is the enzyme that detoxifies lipid hydroperoxides.^[46]^ Loss of GPX4 activity, due to low GSH or direct inhibition, allows lipid peroxidation to proceed unchecked.^[42]^

Lipid peroxidation involving the mitochondria and NADPH oxidases contributes to ROS production. Peroxidized PUFAs in membranes are toxic and disrupt cell integrity. Excess iron (Fe²⁺) also catalyzes lipid peroxidation through the Fenton reaction. Chelation of iron, with DFO, can protect against oxytosis.^[45]^ ACSL4, which enriches membranes with PUFA-containing phospholipids, increased susceptibility to peroxidation.^[39]^ Lipoxygenases (LOXs) are enzymes that oxidize PUFAs in membranes, contributing to the execution phase of oxytosis. Thus, System Xc⁻ inhibition leads to cystine depletion and decreased GSH and GPX4 activity, resulting in increased lipid ROS and membrane damage leading to neuronal death.

### Parthanatos

Parthanatos is a distinct form of regulated cell death triggered by overactivation of poly(ADP-ribose) polymerase-1 (PARP1) in response to severe DNA damage.^[12,47]^ The name comes from PAR (poly [ADP-ribose]) and Thanatos (Greek for “death”). Unlike apoptosis, Parthanatos is caspase-independent and is driven by energy failure and nuclear-mitochondrial signaling involving apoptosis-inducing factor (AIF).^[48]^ The key steps and players in Parthanatos include DNA damage triggered by oxidative stress (ROS/RNS; reactive nitrogen species), alkylating agents, excitotoxicity leading to severe DNA strand breaks, and PARP1 activation.^[49]^ PARP1 normally repairs DNA by synthesizing poly (ADP-ribose) (PAR) from NAD⁺. In overactivation, PARP1 consumes massive amounts of NAD⁺, depleting ATP and leading to metabolic collapse. PAR polymer accumulation is toxic, and PAR can translocate to mitochondria and act as a death signal.

PAR induces mitochondrial outer membrane permeabilization. AIF, normally anchored in the mitochondrial intermembrane space, is released and translocates to the nucleus. The nuclear AIF causes large-scale DNA fragmentation ( ∼ 50 kb fragments; chromatinolysis) and chromatin condensation. This is distinct from the oligonucleosomal fragmentation seen in apoptosis. Other supporting factors include macrophage Migration Inhibitory Factor (MIF), which acts as a nuclease downstream of AIF to promote DNA degradation.^[50]^ DNA histone modifications may influence susceptibility, with oxidative stress as both an initiator and an amplifier. Thus, DNA damage leads to PARP-1 overactivation, NAD⁺/ATP depletion, and PAR accumulation. This results in mitochondrial AIF release and nuclear translocation, resulting in large-scale DNA fragmentation and cell death.

**Figure 2:**
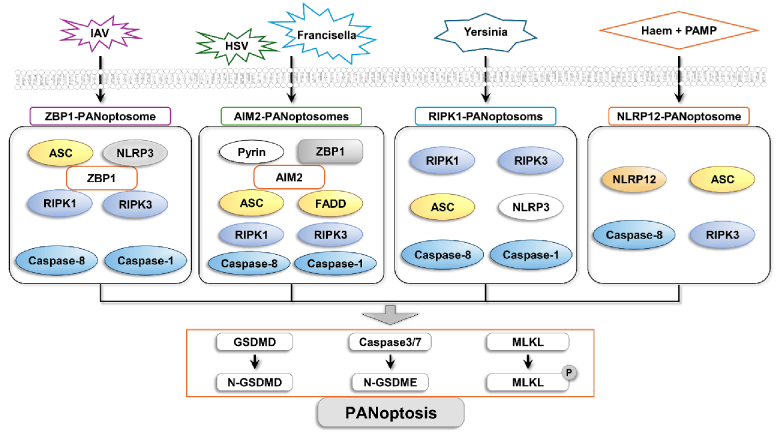
Formation and composition of PANoptosomes. Schematic representation of distinct PANoptosome complexes assembled in response to various pathogenic and inflammatory stimuli. Key inflammasome sensors, including AIM2 (Absent in Melanoma 2), NLRP12 (NOD-like receptor family pyrin domain–containing protein 12), and NLRP3 (NOD-like receptor family pyrin domain-containing protein 3), participate in PANoptosome formation during infections such as influenza A virus (IAV) and herpes simplex virus (HSV). Activation of these complexes coordinates the engagement of multiple cell death pathways, resulting in the cleavage of Gasdermins, including Gasdermin D (GSDMD) and Gasdermin E (GSDME), whose N-terminal fragments (N-GSDMD, N-GSDME) form membrane pores and execute inflammatory cell death. The image was adapted from Jiang et al.^[131]^

### PANoptosis

PANoptosis is a relatively new form of programmed cell death that integrates features of three traditionally distinct pathways, namely pyroptosis, apoptosis, and necroptosis, into a single coordinated process.^[14,15,16][51]^ The term comes from Pyroptosis + Apoptosis + Necroptosis, and the “PAN” also alludes to “all-encompassing” cell death. Unlike individual pathways, PANoptosis is regulated by a multiprotein scaffold called the PANoptosome that assembles in response to certain pathogens or inflammatory stimuli. The four known types of PANoptosomes are shown in Figure 2.

PANoptosome consists of three types of proteins:^[16]^ Sensors, which detect specific stress signals; Adaptors, which facilitate the complex assembly; and Executioners, which directly mediate cell death. The formation of this platform distinguishes PANoptosis from other individual forms of cell death. It is in the context of this platform that the simultaneous activation of the key effectors from all three death pathways, often in response to pathogen infection (influenza virus, SARS-CoV-2, and HSV-1), inflammatory cytokines (TNF and IFN- γ ), and certain cancers or sterile inflammation, is coordinated. Thus, PANoptosis merges components from pyroptotic, apoptotic, and necroptotic signaling cascades through specific assembly of a set of sensors, adaptors, and executioner proteins into PANoptosomes.

The key components of PANoptosomes includes the PANoptosome scaffold proteins (core regulators) including Z-DNA-binding protein 1 (ZBP1; a sensor for viral nucleic acids especially influenza, HSV-1, and a major initiator of PANoptosis), AIM2 (absent in melanoma 2, a DNA sensor that can integrate into PANoptotic complexes), RIPK1 (receptor-interacting protein kinase, scaffolding and signaling in apoptosis and necroptosis), RIPK3 (essential necroptosis kinase part of PANoptosome assembly), ASC (apoptosis-associated speck-like protein containing a CARD, an adaptor protein that bridges inflammasomes to caspases), Caspase-8 (apoptosis initiator caspase, also regulates pyroptotic and necroptotic machinery), and FADD (Fas-associated death domain protein, an apoptotic adaptor protein).^[15,16,51]^ Thus, in PANoptosis, different PAMPs and DAMPs are recognized by specific sensors, initiating the assembly of the PANoptosome, and ultimately initiating apoptosis, necroptosis, and pyroptosis through engagement of appropriate effectors.

The downstream effectors from each of the three pathways are: (1) Pyroptosis arm: Caspase-1 and Gasdermin D (GSDMD, forms membrane pores); (2) Apoptosis arm: Caspase-8 (initiator), Caspase-3, -7 (executioners), and PARP cleavage and DNA fragmentation; and (3) Necroptosis arm: RIPK3– phosphorylates MLKL, which executes necroptosis by disrupting membranes. Elevation of these key PANoptosis proteins related to various regulated cell death pathways with various ocular pathologies supports their participation and cross-communications among these pathways. Cell death signals are diverse and adaptable in response to pathogens and threats. Inhibiting specific proteins can activate multiple regulated cell death pathways, allowing the body to protect itself through alternative mechanisms. While blocking a single pathway may provide partial recovery, addressing multiple pathways, as needed in PANoptosis, where a coordinated network offers a more comprehensive approach to cellular death regulation and more effective disease management.

### Cuproptosis

Cuproptosis is a relatively new form of regulated cell death, which is distinct from those discussed above, and is driven by dysregulation of copper homeostasis. Copper is an essential trace element with a pivotal role in various biological processes, including mitochondrial respiration, antioxidant defense, enzymatic function, and angiogenesis. It is a vital cofactor necessary for the maintenance of biological functions and has been implicated in the development of various types of cancers and metabolic and neurodegenerative disorders.^[52,53,54,55,56,57]^ Thus, restoring copper homeostasis and sensitivity has great potential in the treatment of neurodegenerative diseases and cancer. However, transcriptional reprogramming could influence responses to cuproptosis, and their targeting could be essential to restore their copper sensitivity.^[58]^

Copper homeostasis is tightly regulated by transporters CTR1 and ZnT1, which mediate copper uptake, as well as by intracellular copper chaperons and exporters such as ATP7A and ATP7B.^[59,60,61,62]^ Excessive accumulation of copper can lead to cuproptosis, characterized by direct interaction of copper ions with lipoylated components integral to the mitochondrial tricarboxylic acid (TCA) cycle.^[63,64]^ This binding event triggers the aggregation of these proteins, induces significant proteotoxic stress, and leads to depletion of essential iron–sulfur cluster proteins. Thus, disrupting mitochondrial respiration and increasing inflammation and oxidative stress ultimately culminate in cell death.^[52]^

##  REGULATED CELL DEATH PATHWAYS, OCULAR DISEASES, AND THERAPEUTIC POTENTIALS

Many* in vitro*, *ex vivo,* and preclinical *in vivo* studies have demonstrated the important role of regulated necrosis in various eye pathologies, including ocular surface diseases, glaucoma, retinitis pigmentosa, oxygen-induced ischemic retinopathy (OIR), diabetic retinopathy, and age-related macular degeneration.[9,65-67] Delineating the regulatory mechanisms of these death pathways and their synergistic cross-talks in mediating various blinding diseases will aid in the development of novel therapeutic strategies for more effective management of these diseases. This is currently an active area of research and should advance our understanding of the pathophysiology of various ocular diseases and their effective clinical treatments in the near future.

### Glaucoma

Loss of retinal ganglion cells (RGC) and degeneration of their axons are the ultimate consequence of changes in intraocular pressure (IOP) and optic nerve damage. Previous studies have demonstrated that multiple regulated cell death pathways contribute to RGC loss promoted by mitochondrial dysfunction, oxidative stress, glial cell activation, and excitotoxicity. The important role of necroptosis in the pathogenesis of glaucoma, mainly the loss of RGC cells, has been widely demonstrated in various *in vitro* and* in vivo* preclinical models.^[31,68,69,70,71,72,73]^

Ischemia-reperfusion is commonly used to model the induction of oxidative stress and inflammatory processes that ultimately lead to the death of RGC by necroptosis.^[23,71,74,75]^ This is mediated through the activation of astrocytes and release of TNF- α , which triggers the release of necroptotic factors in the RGC layer.^[68,71]^ Inhibition of necroptosis was shown to be protective in this model.

The expression of Ripk1 and Ripk3 in RGC, with and without ischemia, has been demonstrated. Inhibition of Ripk1 with Nec1 and Ripk3 significantly decreased damage and inflammation after ischemia-reperfusion insult and acute hypertension.^[31,32,71,76]^ Similarly, optic nerve crush-mediated RGC death is mediated by necroptosis, with inhibition of Ripk1 being protective. Thus, necroptosis, a novel form of caspase-independent cell death, contributes to neuronal damage in retinal ischemic-reperfusion injury.

Changes in retinal blood flow could also mediate the necroptosis of RGC, which is protected by inhibition of Ripk1 activity.^[32,68]^ Genetic inactivation of Ripk1 provides protection to RGC, with moderate protective effects from global deletion of Ripk3 and Mlkl, in response to optic nerve crush or ischemia-reperfusion injuries. Knockdown of Ripk1 suppresses microglia infiltration and reduces Ripk3 and TNF- α expression.^[71]^ Administration of anti-inflammatory and antioxidant agents also protects RGC from ischemia-reperfusion injury.^[73,74]^ This may involve decreasing necroptosis in astrocytes and production of inflammatory mediators, including iNOS, Cox-2, and PGE2 receptor.^[74]^

The loss of RGC in glaucoma has been the focus of many early studies, and apoptosis was recognized as a main focus of the death pathway involved. Recent studies, however, indicate a significant role for necroptosis in the loss of RGC in response to ischemia-reperfusion damage.^[23,32,71,76,77]^ However, other forms of programmed cell death may also contribute to the death of RGC cells, and treatments that concurrently target multiple regulated cell death pathways may be most effective. This notion is supported by the lack of a single neuroprotective agent that can preserve the integrity of RGC in glaucoma.^[45,47,75,78]^

Necroptosis in microglia mediates chronic neuroinflammation and degeneration, which is Ripk1-dependent.^[79]^ RGC express Ripk3, which mediates their necroptosis.^[80]^ Ripk3 deletion in microglia mitigates retinal neovascularization through decreased necroptosis and inflammatory processes.^[9]^ Necroptosis of microglia contributes to neuroinflammation and retinal degeneration through TLR4 activation and release of TNF- α and CCL2.^[81]^ A recent study identified 11 modes of cell death in the mouse retina in response to optic nerve injury.^[78]^ Thus, targeting multiple death pathways may be needed to effectively protect against loss of RGC in glaucoma. However, clinical translation of the knowledge gained from these *in vitro* and *in vivo* preclinical studies awaits further clinical exploration.

### Retinal Degeneration

Regulated cell death pathways also play a major role in outer retinal degeneration.^[9,66,79,82,83,84,85,86]^ Photoreceptor cells die by both apoptosis and necroptosis.^[85,87]^ Light damage in photoreceptor cells is mediated by inflammation-mediated necroptosis, leading to the death of rods and cones.^[83,88]^ Suppression of thyroid hormone signaling mitigates necroptotic activity and oxidative stress responses in degenerating retina and enhances cone photoreceptor viability.^[89]^ TNF- α production downstream of neuroinflammation and necroptosis could activate Sarm1 NADase activity, increase calcium influx, and axon degeneration.^[90]^ Anti-TNF- α mitigates retinal degeneration by reducing PARP1 activation, microglia activation, and inflammasome activation without affecting caspase-dependent mechanisms.^[91]^

The light damage induced retinal degeneration could occur through engagement of ferroptosis regulatory pathways.^[85]^ UVA exposure generates ROS that lead to the activation of Ripk3, but not Ripk1, mediating necroptosis through activation of Mlkl.^[92]^ UVA exposure also similarly induces corneal endothelial cell death through necroptosis.^[28]^ Thus, Ripk3 could be a suitable neuroprotective target against oxidant and alkylating agent-mediated injury.^[49,93]^

Mitigation of Ripk1 activity also provides protection in outer retinal degenerative diseases such as AMD and retinal detachment.^[94,95,96]^ Ripk3 deficiency also protects against photoreceptor cell death during the early stages of murine ocular CMV infection.^[97]^ This is attributed to the ability of Ripk3 to activate the inflammasome and NF- κ B, enhancing innate immune responses and promoting cell death by apoptosis and necroptosis. Ripk3-deficiency is protective against alkylation-induced retinal degeneration.^[49]^ Inhibition of apoptosis, by overexpression of XIAP (X-linked inhibitor of apoptosis), protects against photoreceptor loss with retinal detachment.^[98]^ Treatment with Necrox-5, a ROS scavenger and necroptosis inhibitor, protects from retinal degeneration.^[99]^ Thus, different cell death pathways contribute to retinal degeneration, and treatment with antioxidant, anti-inflammatory, and neuroprotective agents could protect against retinal degeneration under various pathological conditions. However, their clinical evaluation remains of great interest and could be most effective in preventing retinal degeneration.

### Ocular Surface Diseases 

A recent programmed cell death pathway referred to as PANoptosis includes integration of apoptosis, necroptosis, and pyroptosis. Coordinated engagement of these pathways plays an important role in homeostasis and stress responses in ocular surface diseases.^[100,101]^ Necroptosis results in enhanced inflammatory processes promoting cell death in ocular surface diseases, including dry eye, keratitis, and corneal alkali burn.^[101]^ Fungal keratitis results in oxidative stress, which initiates Ripk3/Mlkl-mediated corneal epithelium necroptosis and NLRP3 inflammasome signaling through engagement of pyroptosis.^[102]^

Caspase 8 acts as a mediator of the transition between these death pathways. Caspase 8 expression increases following fungal infection and blocks Ripk3/Mlkl-mediated necroptosis. This results in enhanced pyroptosis, inflammasome activation, and release of IL-1 β to provide an early immune protective response.^[36]^ Ripk3-mediated necroptosis also drives macrophage recruitment and corneal neovascularization after alkali burn.^[103]^ Thus, dysregulation of regulated cell death pathways is vital to the pathogenesis of ocular surface diseases, and their clinical targeting could be beneficial for treatment.

### Age-related Macular Degeneration

Oxidative stress and inflammation are also involved in the pathogenesis of AMD. Programmed cell death pathways make a significant contribution to the loss of RPE cells and photoreceptor degeneration in the pathogenesis of AMD. In addition to apoptosis, pyroptosis, necroptosis, and ferroptosis may contribute to AMD pathogenesis. A PANoptosis-like cell death was recently reported in an Amyloid- β (A ${}_{\beta 1-40}$ )-mediated preclinical model of AMD.^[104]^ Thus, targeting these pathways could provide a new modality for AMD treatment.^[105,106]^

Increased oxidative stress, due to lipid ROS in RPE cells through deletion of GPX4 (an important antioxidant enzyme) or oxidizing agents, promoted their loss by necroptosis and ferroptosis.^[39,107,108,109]^ This was mediated by increased Ripk3 and Mlkl activation, and as a result, inactivation of caspase 8, further supporting its role as mediator of necroptosis and ferroptosis pathways. Antioxidants and anti-inflammatory agents, as well as inhibition of thyroid hormone signaling, protect RPE cells from oxidative stress-mediated necroptosis and inflammasome activation.^[110,111,112]^

A role for RPE PANoptosis, mediated by ZBP1, has been recently demonstrated in AMD pathogenesis. Increased ZBP1 expression in mouse or RPE cells treated with NaIO ${}_{3}$ and in eyes from human patients with AMD has been noted. ZBP1 is a sensor of double-stranded DNA, perhaps released as a result of mitochondria and endoplasmic reticulum coupling.^[113]^ The silencing of ZBP1 in RPE cells or mice-protected RPE cells and outer retinal changes mediated by NaIO ${}_{3}$ . These changes were also associated with decreased levels of pMLKL, cleaved GASDMD and caspase 3, and the key executioners of the PANoptosis pathway. However, the potential targeting of this pathway in mitigating AMD-mediated changes awaits clinical evaluation.

The intake of several minerals, including copper, iron, magnesium, and selenium, is associated with a decreased risk of late AMD.^[114]^ Copper is vital for various biological processes.^[115]^ High copper levels stabilize Hif-1 α protein levels under normoxia conditions by reducing its ubiquitination. Hif-1 α promotes the transcription of pyruvate dehydrogenase kinase-1 and-3 (PDK1 and PDK3) and MT2A (metallothionein 2A), which repress the expression of DLAT (dihydrolipoamide S-acetyl transferase) and increase the accumulation of methionine, respectively. Methionine sequesters mitochondrial copper, resulting in resistance to cuproptosis, which under hypoxia could promote angiogenesis.^[116]^ Lower copper levels and decreased levels of copper transporter CTR1 are detected in the RPE/choroid of donor eyes with AMD. In addition, copper is essential for mitochondrial biogenesis in RPE cells.^[117,118]^ Thus, dysregulation of copper homeostasis and cuproptosis-related pathologies in AMD needs further investigation.

### Diabetic Retinopathy

Programmed cell death pathways are involved in neurovascular cell loss as noted in diabetic retinopathy.^[119]^ Neuroinflammation plays an important role in the early stages of diabetic retinopathy, which is mediated by activated microglia undergoing necroptosis driven by Ripk3/MlKL.^[120]^ Decreased expression of Ripk3 in diabetic retina or inhibition of necroptosis reduces microglia activation and release of inflammatory cytokines, mitigating neuroinflammation and restoring visual function.

Inhibition of Ripk3 also protects RGC from high glucose-induced necroptosis.^[121]^ Diabetes is also shown to exacerbate eye infection through altered programmed cell death pathways, such as increased caspase 8 (apoptosis) and Ripk3 (necroptosis).^[122]^ OIR is mediated through necroptosis and enhanced inflammatory responses. Expression of miR124-3p in microglia diminishes necroptosis and inflammatory responses through the regulation of Stat3 and decreases Hif1 α and VEGF expression.^[123]^ PDGF-A, a necroptosis-related gene, whose increased expression in neutrophils mediates the infiltration of immune cells, contributes to the development and progression of diabetic retinopathy.^[124]^ However, the clinical relevance of these observations remains to be investigated.

The increased expression of cGAS-STING in myeloid cells in proliferative diabetic retinopathy, laser-induced choroidal neovascularization (CNV), and OIR triggers activation of Ripk1/Ripk3/Mlkl and their necroptosis, driving neuroinflammation and neurovascular dysfunctions associated with these pathologies.^[120,125,126]^ Thus, inhibition of microglia necroptosis could be anti-angiogenic for eye diseases with a neovascular component, including proliferative diabetic retinopathy. We recently showed that inhibition of Ripk1 and Ripk3 activity mitigates CNV in the mouse laser model of neovascular AMD.^[30]^ The translation of these findings awaits their clinical evaluation in the development and progression of diabetic retinopathy.

**Figure 3:**
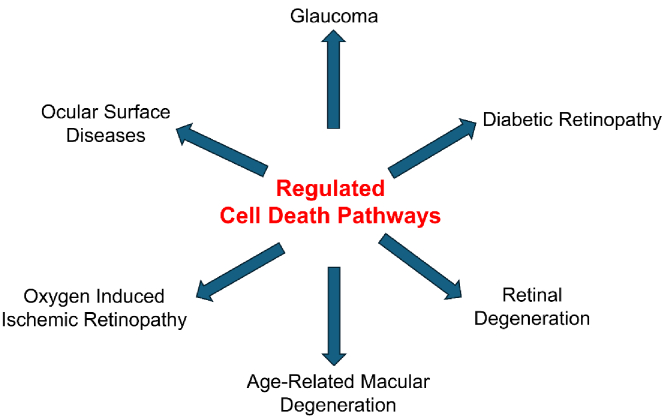
Regulated cell death-related ocular diseases. Regulated cell death pathways are implicated in multiple ocular disorders, including glaucoma, diabetic retinopathy, retinal degeneration, age-related macular degeneration, oxygen-induced ischemic retinopathy, and ocular surface diseases. Understanding the disease-specific pathways and their crosstalk is critical for accurate diagnosis and effective therapeutic development.

The contribution of PANoptosis to complications of diabetes remains incompletely understood. Inhibition of Wnt signaling has been shown to exert a protective effect in diabetic retinopathy, likely by inhibiting PANoptosis and retinal neovascularization.^[127]^ In addition, advances in non-coding RNA research are beginning to unravel their important roles in regulating PANoptosis and the pathogenesis of diabetic complications.^[128]^ More studies are needed to better understand the underlying mechanisms involved and their potential targeting for clinical evaluations.

Elevated levels of copper are also noted in the serum of patients with diabetic retinopathy.^[129]^ Higher levels of copper are also noted in choroid-RPE prepared from human donor eyes with AMD.^[117]^ A recent study also showed an important role for copper-mediated oxidative stress, neurovascular degeneration, and inflammation during ischemia-reperfusion retinal injury utilizing copper transporter Ctr1-deficient mice.^[62]^ Thus, retinal cell death is a prominent feature of diabetic retinopathy, and unraveling the association between regulated cell death pathways and the pathogenesis of diabetic retinopathy will aid in the development of effective treatments for clinical evaluation.

Disruption of copper homeostasis during diabetes contributes to the development of diabetic cataract. Incubation of human lens epithelial cells under high-glucose conditions resulted in increased intracellular copper levels and reduced cell proliferation. These changes were mediated through increased levels of SLC31A1, a cuproptosis-related gene mediating influx of copper ions into the cells.^[130]^ Thus, dysregulation of copper homeostasis through modulation of SLC31A1 during diabetes could mediate cuproptosis and formation of diabetic cataract.

Collectively, the studies presented here demonstrate the progress that has been made in the study of different programmed cell death pathways and their potential contribution to the pathophysiology of various vision-treating eye diseases. Although the results of preclinical studies targeting specific death pathways are promising, more work is needed to better understand the potential involvement of multiple death pathways and their coordinated engagements in various ocular pathologies. This knowledge will help not only help to better understand the pathophysiology of the disease but also more effectively manage it.

##  SUMMARY

Regulated cell death pathways are fundamental to maintaining normal development and tissue homeostasis. Their dysregulation can result in excessive cell death and inflammation, contributing to the onset and progression of numerous ocular diseases [Figure 3]. Increasing evidence suggests that multiple cell death pathways may be activated in a coordinated, tissue- and stress-specific manner to drive distinct pathophysiological outcomes. Unfortunately, there remains a significant lack in our knowledge regarding the clinical relevance of these changes across various eye diseases. Elucidating the intricate interplay and crosstalk among these pathways will not only advance our understanding of disease mechanisms but also uncover combinatorial vulnerabilities that can be therapeutically targeted. Such integrative approaches hold significant translational potential for the development of more effective and durable treatment strategies in the future.

##  Financial Support and Sponsorship

The work in NS laboratory was supported by an unrestricted award from Research to Prevent Blindness to the Department of Ophthalmology and Visual Sciences, Research to Prevent Blindness Steine Innovation Award, Retina Research Foundation, Arthur and Nancy Nesbit AMD fund, Reeves Foundation, The Edward N. & Della L. Thome Memorial Foundation, and by the National Institutes of Health grants P30 EY016665 and P30 CA014520.

##  Conflict of Interest

None.
